# Stock market comovements among Asian emerging economies: A wavelet-based approach

**DOI:** 10.1371/journal.pone.0240472

**Published:** 2020-10-12

**Authors:** Ijaz Younis, Cheng Longsheng, Muhammad Farhan Basheer, Ahmed Shafique Joyo

**Affiliations:** 1 School of Economics and Management, Nanjing University of Science and Technology, Nanjing, PR China; 2 School of Economics Finance and Banking (SEFB), Universiti Utara Malaysia (UUM), Sintok, Malaysia; 3 Department of Business Administration, Shaheed Benazir Bhutto University, Shaheed Benazirabad, Pakistan; The Bucharest University of Economic Studies, ROMANIA

## Abstract

Stock market, is one of the most important financial market which has a close relationship with a country’s economy, due to which it is often called the barometer of the economy. Over the past 25 years, the stock markets have been affected by different global economic shocks. Various researchers have analyzed different aspects of these effects one by one, however, this study is an assessment of stock market interrelationship of emeriging Asian economies which include most of the East Asian, and Southeast Asian emerging economies with special focus on China for past decades during which different crisis occurred. We used Morgan Stanley capital international (MSCI) daily indices data for each stock market and compared Chinese stock market with the stock markets of India, Pakistan, Malaysia, Singapore, and Indonesia. We analyzed the data through the individual wavelet power spectrum, cross-wavelet transform and wavelet coherence, to determine the correlation and volatility among the selected stock markets. These model have the power to analyze co-movements among these countries with respect to both frequency and time spaces. Our findings show that there are co-movement patterns of higher frequencies during the crises periods of 1997, 2008 and 2015. The dependency strength among the considered economies is noted to increase in the crisis periods, which implies increased short- and long-term benefits for the investors. From a financial point of view, it has been determined that the co-movement strength among the emerging economies of Asia may have an effect on the VaR (Value at Risk) levels of a multi-country portfolio. Furthermore, the stock market of China shows a high correlation with the other six Asian stock emerging markets in both high and low-frequency spectrums. The association of the south and east Asian stock market with Chinese stock markets show the interconnection of these economies with the economy of China since past two decades. These findings are useful for investors, portfolio managers and the policymaker around the globe.

## 1. Introduction

The stock market is one of the major components of the financial market which is interconnected to the real economic operations. It meets the financial needs of the corporate sector while giving investors a chance to earn from the trading of the stocks. The stock markets are considered the barometers of the economies. They get affected by economic fundamentals and the global economic shocks [1]. With globalization, the international capital flow among the markets has increased, which make different markets affect each other’s performance [2–4]. Since the late 1990s, the global economies have been affected by the economic crisis which resulted in increased co-movements among the stock markets around the globe [5]. The change in the stock market comovement has changed the investing strategies of the portfolio investors around the world, thus making the stock market interconnection a subject of interest for the researchers. Many pieces of research have analyzed the stock market movements giving special reference to Mexican peso-devaluation (1994), the East Asian financial crisis (1997), global financial crisis (2009) and so on, which can be found widely in the literature [2–4, 6]. Besides the individual researchers the global economic and monitory institutions like the International Monetary Fund (IMF), Banks for International Settlements (BIS), Financial Stability Board (FSB) and other regulatory authorities have studied the nexus of the international stock markets with the standpoint of the systemic risk which was underestimated across the board before the crisis. These studies unanimously found the disorder in the local markets as the initial cause of the crisis, which leads to market excess in the volatility and extreme interconnection between the stock markets [7]. Most of the past researches have focused on the developed market and found a high level of interconnection among the developed market with little diversification opportunities. However, there is an increase in the studies on the emerging market in a quest of diversification opportunities among the emerging markets.

As after China-US trade, the focus of the trade and investment have changed to the east Asian emerging markets [8, 9]. Therefore, in this study, we have focused on the Asian emerging economies keeping China at the center. Another reason for choosing China and other Asian emerging markets is the regional proximity. The growing body of literature has derived many theoretical justifications explaining the stock market correlation such as the theory of stock market comovement and the stock market interdependence based on the law of one price (LOP) and Modern portfolio theory (MPT) [2–4]. These theories inculcate to diversify the portfolio among the international markets [6, 10, 11]. The investor’s expectation theory claims that investors similar expectation are true reflectors of their investments.

The underlying principle of capital asset pricing theory and arbitrage pricing theory, which emphasizes that international markets are correlated, and there is the same price of a unit of a commodity in all the international markets. The stock market efficiency theory assumes that the flow of stock prices information creates a correlation among the international stock markets. The behavioral finance theory presumes that the investor preferences based on certain subjective factors create a herding effect which correlate the stock markets. Information spillovers effect like the stock market efficiency theory takes the information of the stock market as key determinants of stock market correlation. However in addition, it assumes that the spillover of stock information among the regions and countries with varying time zones as a reason why the stock information correlates the international stock markets The measure of stock market comovement has become a yardstick to analyse the portfolio diversification benefits for the investors as well as the resulting benefits to the real economy in terms of economic growth and connectedness with the global markets. The higher comovement among the stock market shows that the selected economies are highly interdependent, moving in the same direction with the global economies while the low comovement shows that there is an opportunity for the stock market investor and the diversification opportunities exist. International portfolio-diversification can be a tool for portfolio efficiency improvement due to the resulting reduction of risk [12–14].

There has been burgeoning research on the stock market linkage to understand the market behavior and the investment opportunities using stock market data. The past researchers have used different tools like Correlation coefficient, GARCH models, and Copula models [79]. Besides these models, Wavelet model has proved an appropriate alternative to other time series models. Wavelets-based models proposed by [15, 16] have emerged as a dynamic tool in different scientific applications, such as medicine [17–19], astrophysics [20–23] and geophysics [24–26]. Particularly financial markets research, the variants of wavelets model like continuous wavelet transform (CWT), wavelet coherence (WCOH), cross wavelets transformation (XWT), and wavelet correlation analysis (WCA) have been used to analyze the co-movements between the pairs of the stock markets [27–29]. Wavelets based methods have various advantages compared to Fourier analysis. These models capture the time dynamics over different frequencies and are presented in the form of color spectrums [28, 29]. state that WCOH, has more capability of identifying high co-movement regions in a time and frequency space as compared to the wavelet correlation analysis.

This research studies the co-movement among various stock price-indices of the emerging market of Asia based on the wavelet approach. We selected six emerging economies, including China, India, Indonesia, Malaysia, Pakistan, and Singapore. We used daily stock price data from MSCI indices from 1993 to 2019. This time period covers three crisis periods: Asian economic crisis 1997–98; subprime mortgage caused global crisis 2007–09, and the crash of the stock market in China 2015. In the first step, we described each time series with the time series graph, descriptive statistics, and other preliminary tests. In the second step, we used individual power spectrum to analyses individual series variance [30–32], we refer to cross wavelets transformation (XWT) to show the local covariance between time-series, as well as the wavelet coherence (WCOH) to examine the co-movements between the pairs of each selected with Chinese stock markets. In the third step, we used the VaR (Value at Risk) of the portfolio of the Asian emerging markets. In all, the wavelet approach is useful to capture the actual situation in the Chinese stock market with its emerging economies of south Asia, which justifies the usefulness, suitability and robustness of our method [6].

This research brings valuable insight for the investors who want to diversify their investments to emerging international stock markets. It is evident that the investors favor high volatility stocks in normal periods, whereas they prefer low volatility stocks to stay safe in the periods of crisis [7]. However, in this research, we have a combination of the Asian emerging markets with china which could bring a result of great interest for the investors. Further, this study brings insight into the policymakers in order to increase the integration of the Asian markets with China while avoiding the risk of contagion [33]. On the financial side, we uncover that the strength of co-movement among Asian emerging markets may impact the multi-country portfolio's value at risk (VaR) levels. These findings provide potential implications for portfolio managers operating in the Asian region who are invited to consider co-movement through both frequencies and time when designing their portfolios.

The rest of the paper is organized as follows; in section 2, a brief literature review is provided; in section 3, methodology and data are discussed; section 4 discusses the models for identification of the driving sources and discussion of the results, and finally, the work is concluded in section 5.

## 2. Related literature and research motivations

The literature on the stock market relationship has many dimensions in terms of empirical methodology, scope and selection of Stock markets. However, in this section, we have focused on the empirical literature review focusing on the studies from different parts of the world using different parametric and non-parametric techniques. Aloui and Hkiri [6], used a squared coherence wavelet approach to examine the relationships among the returns of GCC (Gulf Cooperation Council) stock markets. They showed that strong dependencies exist among the considered markets, especially during the recession of 2007. Another study by Graham, Kiviaho [34] used the wavelet coherence approach, where the local and global trends in co-movement from the stock markets of the MENA region. It was found that the co-movement in these markets varied across frequency and over time, and a strong coherence was detected during the recession of 2007. The frequency-domain showed higher dependencies in the lower frequency regions as compared to the high-frequency region meaning that the coherency is more pronounced in the long-run. Similarly, Loh [35], studied the interrelation among stock markets of the Asia-Pacific region against the stock markets of US and European regions through coherence approach of wavelet analysis. They considered 13 markets from Asia-Pacific region and showed considerable co-movement variations during the periods of recession, it was found, as well, that over the medium-scale, higher coherence existed in the sub-prime period of recession as compared to the low-scale during the Debt crisis in Europe.

The stock markets from the GCC region were studied by Akoum, Graham [36]. In this study, dependencies between oil price trends and stock market were observed at the multi-scale level. It was found that although strong dependencies existed at the low-frequency domain, its strength varied across the considered countries. Graham, Kiviaho [37] examined the coherency among 22 emerging markets around the world with the US stock market. In case of low frequency, it was found that high coherency existed, with a slight shift of trend in the year 2006, where the coherence was especially found in the high-frequency domain. The relationship among the stock exchanges, FTSE100, DJIA30, Nikkei225 and Bovespa, was examined through wavelet coherence analysis by Madaleno, Pinho [38] where a strong coherence was observed in lower frequency zones. Rua, Nunes [39] studied in terms of time-frequency through continuous wavelet transform model, the systematic risk was found to be significant, and it was determined that its importance was comparatively higher and more stable in lower frequency zones.

The relationship between management priorities, on hedging horizon, expected the return and risk-aversion rate, and their aggregate effects on hedging efficacy were analyzed by Conlon, Cotter [40], and it was found that with a change in hedge horizon, there was a significant variation in hedge ratio. Further, best risk-reduction was observed for longer horizons in case of high-risk investors, whereas low-risk investors were more compatible with shorter horizons. In a comprehensive study by Conlon and Cotter [41], the efficacy of hedging in different ranges of hedge horizons was explored through wavelet transform tool, and as a result, the concept of ‘‘time diversification” was verified. Masih, Alzahrani [42], used wavelet-based techniques to analyze systematic risk variation, for the financial GCC (Gulf Cooperation Council) it was determined that VaR (Value at Risk) is comparatively more intense at higher frequencies. Trimech, Kortas [43] utilized wavelet models by combining it with the Fama–French three-factor model. It was found that the relationship between risk and portfolio returns was dependent on the range of investment. This finding highlights that the impact of investment range shifts on the risk-aversion rate among the French stock markets. Similarly, hedge fund scaling properties against S&P 500 were measured through MODWT (Maximum-Overlap Discrete Wavelet Transform) by Conlon, Crane [44], where it was shown that the rate of risk changes with change in investment range.

The effect of the sub-prime crisis of 2007–08 was analyzed in a study by Benhmad [5], where the change in stock market correlation was studied. The study revealed that various factors, including market conditions and local features, determine the dependency strength. In another study, In and Kim [45] used wavelet correlation method to study the relationship between the stock market and futures market. It was revealed that the correlation in wavelet had a significant variation on the investment scale. Kim and In [46] used another application wavelet correlation method, where the stock return-inflation relationship was analyzed, and it was concluded that a positive relationship existed at the extreme time-scale, whereas negative relationship was found at the moderate scale. Similarly, the wavelet method was used to analyze the industrial levels of output from the countries in G-7 [47], and the study revealed strong scale-dependencies among these countries.

Employing the data of daily stock indexes over the period of six years from October 1992 to June 1997, Huang, Yang, and Hu [48], found that there is no cointegration relationship between the mainland Chinese stock market and the markets Hong Kong, Taiwan, Japan and the US. Later the study of Hsiao, and Yamashitac [49], shown consistency with the findings of Huang, Yang, and Hu [48] and concluded that any abrupt change in the US market would not bring any significant change mainland Chinese market. However, one of the limitations of these studies that they have used earlier data. Later Lin, Menkveld, and Yang [50] found that, during the period from 1992 to 2006, the Chinese markets are correlated with the major western markets. Lai and Tseng [51] carried out a study explaining the co-movements of Chinese and G7 market, and their results reveal that the Chinese stock market and G7 stock markets. Wang, Chen, and Huang [52] found that the Chinese market is heavily dependent upon the Asian pacific market.

Though a number of studies have been carried out on the issues related to the integration of stock markets in the lading equity markets [53–57]. However, during the course of recent decades, the increasing attention has been given on the exploration stock market integrations among the Asian markets. The first group of researchers [58–61] started with the examination of the integration of key Asian markets such as Japan with world-leading economies such as the USA. In continuation of this Chung and Liu [62] found that the East Asian markets are cointegrated with US and Japanese markets. However, the study concluded that during the period from 1986 to 1992 the cointegration of east Asian stock markets with US were stronger than that of Japan. Later on, Arshanapalli et al. [63] also confirmed the findings of Chung and Liu [62] provided the evidence of dynamic linkages of Hong Kong, Philippines, Malaysia, Singapore and Thailand with the USA and Japan during 1986–1992. Darrat and Zhong [64] studied the long-term co-integration between the 11 Asia specific markets with US and Japan over the period of 13 years from 1987 to 1999, and found that Asian markets are co-integrated with US stock market. Darrat and Zhong [64] investigated the long-run relationship between 11 Asia-Pacific markets with the USA during 1987–1999.

Apart from that, there are studies which examined the co-integration among Asian markets. For example, Hung and Cheung [65] found that the Asian equity markets namely the equity markets of Hong Kong, Malaysia, South Korea, Singapore and Taiwan are co-integrated Manning [66] examined market integration among Hong Kong, Indonesia, Japan, South Korea, Malaysia, Philippines, Singapore and Thailand during 1988–1999. Recently, Chien et al. [67] claimed that the ASEAN markets are progressing toward financial integration. However, Hee Ng [68] indicated the absence of a long-run relationship among ASEAN countries during 1988–1997. It can be observed that literature has mixed evidence on stock market integration. This may be because different studies have examined the different set of markets in different time periods and have employed different methodologies.

There has been an expeditious development in Chinese economy due to which its stock market has attracted the investors, and academicians to grasp the understanding of the uniqueness of Chinese stock market, and how it is linked with the regional and international markets. A separate strand of literature has studied the correlation between the stock market of China with the stock market of the other countries such as Asia-Pacific, Greater China, Japan, the United States and the United Kingdom [69–73]. The earlier studies have reported that there is weak or no correlation between the stock market in China with the stock markets in other economies [48, 69]. However, later a group of researchers have found that the correlation between the Chinese market and the international markets has increased over time [69, 74]. We, therefore, probe into this neglected issue while focusing on the Chinese, and emerging Asian markets to fill the void in the relevant literature.

The literature related to stock market connectedness using various aspects of the Fourier based wavelet mythology is increasing day by day. These studies have covered various regions and economic blocks from all over the world. Most of these studies have covered fever years, and focus has been the developed stock markets. However, with the changing global dynamics, the need for news studies is felt. We feel that there is still a need for a study which should address the interrelationship of the south and east Asian stock markets with China over the extended period of time. Therefore, this study contributes to the existing literature by studying the stock market relationship of Asian emerging with China over the last twenty-five-year period.

## 3. Materials and methods

### 3.1 Data and preliminary analysis

This study uses the Stock indices data of six emerging countries comprising of China, and its major trading partners, namely India, Indonesia, Pakistan, Malaysia and Singapore over 25 years. We collected the daily data of MSCI indices for these countries from DataStream database for the period starting from January 1993 to August 2019. Especially, the considered data cover most important stock crises dates (1997–98, 2007–08 and 2015). In these periods, the tensions in the global markets were recorded to be high in June 1997, while the impact from financial turmoil was intensified in 1998 as a result of Russia's financial instability. However, these effects faded by March 1999. The recovery phase is considered to be 1^st^ March 1991 to 31^st^ Dec 2002.

It is worth mentioning that the considered sample period represents some important events from an economic point of view. They include the rise of oil prices in 1993 and 2019, the crisis of Mexican currency (1994–95), Asian economic crisis (1997), Russian default (1998), Argentine crisis (1999–01), the stock crisis of Brazil (1997–98), US sub-prime crisis (1998), world economic collapse (2008–09) and its slow recovery in 2010. The Shanghai stock market collapsed from the peak recorded on 12^th^ June 2015 to the bottom on 8^th^ July 2015. This collapse wiped out around 18 Trillian yuan of the value of shares, losing 32% of its composite indices [75, 76]. Our study needs only to consider the daily changes in prices [77], and non-stationary behavior of the stock markets cannot be used with wavelet framework which makes filtering of data, not a necessary act. Therefore, only daily indices of the market price are used instead of levels.

### 3.2 Methodology

We use a wavelet approach to determine the interrelationship between the selected stock markets. Wavelet has a strong potential to capture the non-stationary behavior and time-varying trends present in the stock price data [6, 30, 31]. Different studies provided a detailed and formal description of the wavelet approach [15, 29]. In this sub-section, we briefly expose the wavelet as a suitable tool for analyzing the Asian emerging stock market co-movements in the frequency-time domain. In this study, we discuss the Individual power spectrum, cross wavelet transforms and wavelet coherence for examining the relationships between two-time series. In addition, this research shows how phase angle statistics can be used to gain confidence in causal relationships and test mechanistic models of physical relationships between the corresponding time series. Monte Carlo methods are used to assess the statistical significance against red noise backgrounds. Further, wavelets provide more precise timing of shocks which cause a change in interrelationships between time series.

#### 3.2.1 The wavelet

The wavelet is a technique used for time series behavior assessment with respect to both time and frequency domain. In this work, wavelet is employed as specified by Morlet’s for co-movement assessment of emerging Asian market due to its adaptability. The method of wavelet helps to decompose a time-series (*ψ*_*u*,_ (*t*)) into components form, which allows us to read the time-series information. Using the same notations as in [6, 30, 31], and in earlier works, by [15], and [26] we define wavelets as:

$$\psi |t|=\frac{1}{\sqrt{s}}\psi |\frac{t-u}{s}|$$ (1)

Firstly, one should recall that a wavelet is a real-valued or a complex-valued function *ψ*(.) defined over the real axis. It is also assumed that *ψ*(⋅) ∈ *L*^2^(ℝ), i.e., the wavelet is assumed to be a square-integrable function. Here $1/\sqrt{s}$ represents the factor of normalization ensuring that ${\left\|\psi_{u,s}\right\|}^{2}=1$, where u represents the position of the respective wavelet and s represents the parameter for dilation of scale (defines how the wavelet is scaled, i.e., stretched or compressed). The wavelet specified by Morlet is defined as:

$$\psi_{0}^{M}|t|=\pi^{-1/4}e^{i\omega_{0}t}e^{\frac{{-t}^{2}}{2}}$$ (2)

here *ω*_0_ represents the wavelet’s central frequency, and according to [26, 29] and [78]. This choice of value for *ω*_0_ enables a good balance between time and frequency localizations.

#### 3.2.2 Continuous wavelets

Following [29] and [78] the continuous wavelet transform can be described as:

$$W_{x}|u,s|=\int_{-\infty }^{\infty }x(t)\frac{1}{\sqrt{s}}\overset{¯}{\psi |\frac{t-u}{s}|dt}$$ (3)

here *W*_*x*_(*u*,*s*) can be found through a *ψ*(.) projection on the time-series, where *ψ*(.) denotes the specific wavelet. The ability of CWT to decompose and combine the function *x*(*t*) ∈ *L*^2^(ℝ) is an advantage such that

$$x|t|=\frac{1}{c_{\psi }}\int_{0}^{\infty }\left[\int_{-\infty }^{\infty }W_{x}(u,s)\psi_{u,s}(t)d_{u}\right]\frac{d_{s}}{S^{2}},\;s>0$$ (4)

The time-series preservation of wavelet transformation is its unique feature. The variance for the power spectrum can be specified as follows.

$$x|t|=\frac{1}{c_{\psi }}\int_{0}^{\infty }\left[\int_{-\infty }^{\infty }|W_{x}{\left(u,s\right)}^{2}|d_{u}\right]\frac{d_{s}}{S^{2}},$$ (5)

#### 3.2.3 Wavelet power spectrum

Similarly, the power spectrum for wavelet is described as ${\left|W_{nx}\right|}^{2}$ and it can simply evaluate the local variance for each variable [28]. In addition, the importance in terms of statistics is assessed when the comparison is made with the default hypothesis. As demonstrated in [15] through the simulations of Monte-Carlo white- and red-noise can be utilized at each time n and space s instance, and to obtain the corresponding distribution for the local wavelet power spectrum such that:

D(|Wnx(s)2|Xσ2<p)⟹12PfX2v (6)

where *P*_*f*_ is the mean of the spectrum at the Fourier frequency *f* that corresponds to the wavelet scale *s*(*s* ≈ 1/*f*), and *v* takes the values of 1 or 2 for real or complex wavelets.

#### 3.2.4 Cross-wavelet transform, wavelet coherence and phase differences

Neighborhood covariance of two-period series is preserved in all frequencies by the Cross-Wavelet Power (hereafter., XWP). In this work, XWP is used to locate the high market price-co-movement regions in case of time-frequency domain [28]. The two-signal cross-wavelet can be defined through the spectrum of cross-wavelet ($W_{n}^{XY}(s)$), given as

$$W_{n}^{XY}|s|=W_{n}^{X}(s)W_{n}^{Y^{*}}(s)$$ (7)

here $W_{n}^{Y*}(s)$ represents the complex conjugate of $W_{n}^{X}(s)$. $W_{n}^{XY}|s|,$ the CWP helps to acquire the covariance of the variables. The theoretical distribution of the cross-wavelet power of two signals with power spectra $P_{\kappa }^{X}$ and $P_{\kappa }^{Y}$ is given in the following form:

D(|WnX(s)WnY*(s)|XYσσ<p)=Zv(p)vPKXPKY (8)

where ^*σ*^*X* and ^*σ*^*Y* denotes the standard deviations of *x* and *y*. *Z*_*v*_(*p*) represents the confidence interval, where p denotes the probability for a *pdf* (probability density function), which is defined as the root squared *Χ*^2^ distribution. The wavelet coherence is computed as the squared absolute value of the smoothed cross-wavelet spectra normalized by the product of the smoothed individual wavelet power spectra of each time series specifies the wavelet coherence, given as in [16]

$$R^{2}|u,s|=\frac{{\left|S(s^{-1}W_{xy}(u,s))\right|}^{2}}{S(s^{-1}{|W_{x}|u,s|}^{2}).S(s^{-1}{|W_{y}|u,s|}^{2})},$$ (9)

In the above equation, the smoothing parameter is denoted by *S*. The coefficient of squared wavelet-coherence (CSWC) satisfies the inequality condition of 0 ≤ *R*^2^(*u*,*s*) ≤ 1. A value of *R*^2^(*u*,*s*) approaches one signifies a high correlation, and it suggests correlation is considered to be weak if *R*^2^(*u*,*s*) approaches to zero. Due to the above reasons, the wavelet-coherence approach is considered to be the most appropriate method for variable inspection with respect to time and frequency. In addition to this, the two time-series phase-difference variables *i*.*e* *Φ*_*x*,*y*_ can be used to distinguish between their phase-relationship. The phase-difference defined below determines the positions in the pseudo-cycle, and it is specified as:

$$\phi_{x,y}={tan}^{-1}\left(\frac{T\left\{W_{n}^{xy}\right\}}{R\left\{W_{n}^{xy}\right\}}\right)\;with\;\phi_{x,y}\in \left[-\pi ,\pi \right]$$ (10)

The arrow directions characterize the phase relationship. In the event that arrows directed to the right, it suggests that the two variables are positively correlated, and vice versa. In addition, if arrows approach the right and up, the variable x is leading, and the two variables are positively correlated; or if arrows approach the right and down, the variable x is lagging, and the two variables are positively correlated. On the other hand, if the arrows move to the left and up, the first variable x is lagging, and the correlation is negative, or if the arrows move to the left and down, the first variable x is leading, and the correlation is negative.

## 4. Results and discussion

### 4.1 Summary of statistics

The descriptive statistics for the price indices of the stock market are provided in Table 1. The highest and lowest average returns are 3.256401 and 1.684537, which represent Indonesia and China indices, respectively. The highest and lowest standard deviation, in the stock markets, is found to be 0. 434282 and 0.119035, for Indonesia and Singapore stock markets indices, respectively. The stock index for five out of six considered countries is negatively skewed, which shows their poor performance. Indonesia is the only market found to be positively skewed, and hence it shows positive performance during the considered time. The kurtosis for all the countries is found to be less than 3, which indicates that none of the data is distributed normally. The null hypothesis can be rejected with a 1 per cent significance level of the Jarque–Bera test.

**Table 1:** Descriptive statistics of Asian countries.

Description	CHINA	INDIA	INDONESIA	MALASIA	PAKISTAN	SINGAPORE
Mean	1.684537	2.550454	3.256401	2.597011	2.362668	3.113607
Median	1.771227	2.576239	3.242813	2.595090	2.425458	3.122171
Maximum	2.136787	3.141503	3.893184	2.825716	2.815639	3.346083
Minimum	1.111699	1.890276	2.447220	1.945828	1.735383	2.706057
Std. Dev.	0.230425	0.380279	0.434282	0.164337	0.277283	0.119.35
Skewness	-0.76143	-0.00716	0.019106	-0.500412	-0.254222	-0.546260
Kurtosis	2.566887	1.409711	1.374339	2.623771	1.781314	2.574919
Jarque-Bera	726.5360	733.0534	766.3867	331.3370	505.3857	398.3164
Probability	0.000000	0.000000	0.000000	0.000000	0.000000	0.000000
Sum	11717.64	17740.96	22651.25	18064.81	16434.72	21658.25
Sum. Sq. Dev.	369.2803	1005.779	1311.717	187.8303	534.7429	98.54843
Observation	6956	6956	6956	6956	6956	6956

### 4.2 Dynamics of daily stock prices and returns

**Fig 1:**
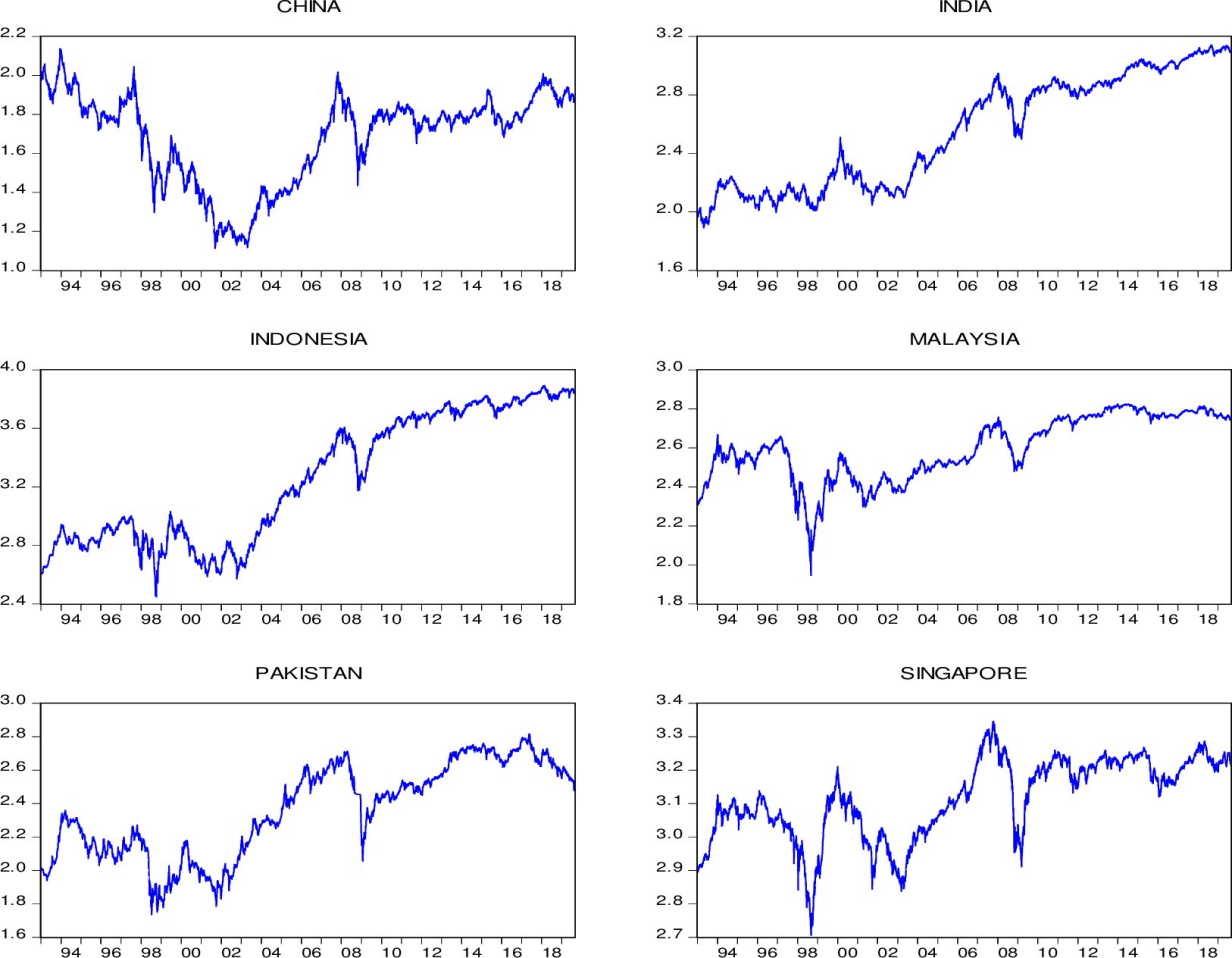
Dynamics of daily stock prices.

The stock price data time-series graphs are presented in Fig 1. It shall be noted that the mean and variance of the data fluctuate across the period 1^st^ Jan 1993 to 30^th^ Aug. 2019. The response to GFC in 2008 can be noted for all markets with a simultaneous decrease. The year 2015 shows declining trends in the Chinese market. This decline is observed due to shortcomings in public policy and gaps in security trading [76]. A 70% fall in the Chinese stock index was observed through this crisis. The Shanghai stock market collapsed from the peak recorded on 12^th^ June 2015 to the bottom on 8^th^ July 2015. This collapse wiped out around 18 Trillion yuan of the value of shares, losing 32% of its composite index [75, 76].

Fig 1 represents the time-series graphs. It can be seen that the return series graph shows mean-reverting trends, and persistence of volatility can also be observed. This type of volatility persistence is often referred to as volatility clustering. The high fluctuations during the financial crisis caused high volatility during 2008. However, the Malaysian market shows lower volatility during GFC. A stationary period around 2008 can be observed on Pakistan’s plot. This stagnant period is due to the rigid floor conditions of KSE (Karachi Stock Exchange), which was held fixed for a period of 110 days by the management. However, the index declined to 4782 points when the floor was lifted [79]. It is perceived that all markets exhibited different trends in this period. For instance, Indonesia, India and Singapore had a slightly higher trend as compared to the other markets. This variation among the countries indicates that the correlation among these markets is low or none.

### 4.3 Correlation of Asian emerging market

The results of the stock return correlation matrix are illustrated in Table 2. Although the positive values for all the selected countries indicate that the markets move in the same direction, the stock markets correlation is found to be within the range 0.4172 to 0.91. Couples with a correlation higher than 0.8 are considered to have a high co-movement. China-India stock market correlation is found be the lowest, while Indonesia-Pakistan stock market correlation is recorded as the highest. It shall be noted that Pearson correlation that does not record any of the variations within the period has been used in this study [79].

**Table 2:** Correlation analysis (emerging market).

COUNTRIES	CHINA	INDIA	INDONESIA	MALAYSIA	PAKISTAN	SINGAPORE
CHINA	1					
INDIA	0.4172	1				
INDONESIA	0.5204	0.9746	1			
MALAYSIA	0.6190	0.8569	0.9060	1		
PAKISTAN	0.4872	0.9086	0.9100	0.8726	1	
SINGAPORE	0.5835	0.8444	0.8515	0.8974	0.8244	1

### 4.4 Individual power spectrum: Emerging market

**Fig 2:**
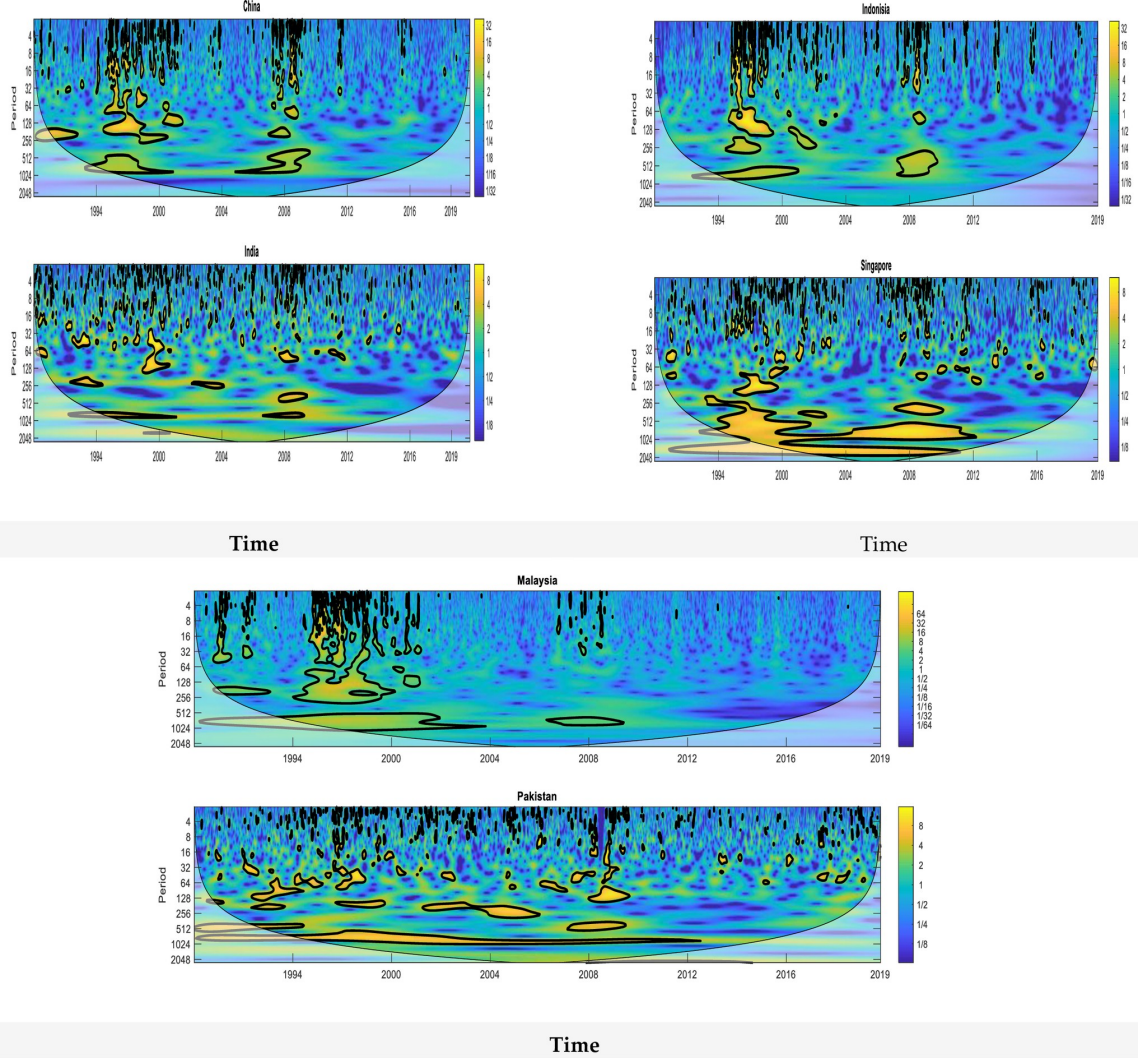
Wavelet power spectra of emerging stock market. The color code for power ranges from dark blue (low power) to dark red (high power). A black thick contour encloses regions of 5% cumulative area wise significance Time (year) and frequency (period) are represented on the horizontal and the vertical axis, respectively.

A three-dimensional contour-plot of wavelet power (hereafter, WP) spectra of the considered stock markets is illustrated in Fig 2. WP spectrum displays the local market variance evolution with respect to the frequency-time domain, where a larger variance is indicated by higher intensity spectra. In Fig 2, the frequency scale is represented by the vertical axis-, the time scale is presented on the horizontal axis, while the intensity is presented on the third scale in the form of colour (blue to yellow colour; low to high intensity). This representation means that across the horizontal-axis (when wavelet scaling is kept constant) and one reads the variation in intensity over time-scale, whereas, down the vertical-axis (when the time scale is kept constant), one reads the variation in intensity over the wavelet-scale. The null-hypothesis of a steady operation is compared with WP spectrum to determine the statistical significance of the WP.

This is achieved by Monte-Carlo simulations through a phase randomized surrogate series [26, 29, 36, 80, 81] The WP spectrum significance level of 5% is represented by the hardy black contour. These edge effects impact the transform and the region of this impact is termed as “COI (cone of influence)”, which is defined as the region where the attained results are unreliable [15, 82].

The cone of influence (hereafter, COI), represented by the lighter shade, apparently separates the high-intensity regions from the lower-intensity regions. It can be seen in Fig 2 that there exists some similarity in the low-frequency WP concentration (corresponding to high scales). It shall be noted that at low frequency (128–256), the spectra of all countries are localized, where high intensity can be seen in China, India, Malaysia, Pakistan and Indonesia in the period 1993 and 1997–1998. All the countries’ stock market indices except Malaysia are localized in the period 2007–08 at medium scales. At high scales of 512–1024, the stock market indices of different countries are found to be localized with high intensities in different periods, which are given for China, India 1994–1998 and 2007–09; Indonesia 1994–2001 and 2007–07, Malaysia 1993–2001 and 2007–08; and Pakistan, Singapore 1993–2011 and 2015.

Generally, except for Malaysia and India, share the same risk pattern over the sample period and across medium-scale bands. From these plots, the riskiest market is corresponding to the Singapore market where a big island of yellow color is scattered over the sub-period 1994–2012. This suggests that the global financial crises in recent times have had high impacts on the emerging markets of Asia. Generally, the existence of localized variation over the same period in the considered stock markets implies the existence of co-movement in some scale and over time.

### 4.5 Cross wavelet transformation: China vs. emerging markets

**Fig 3:**
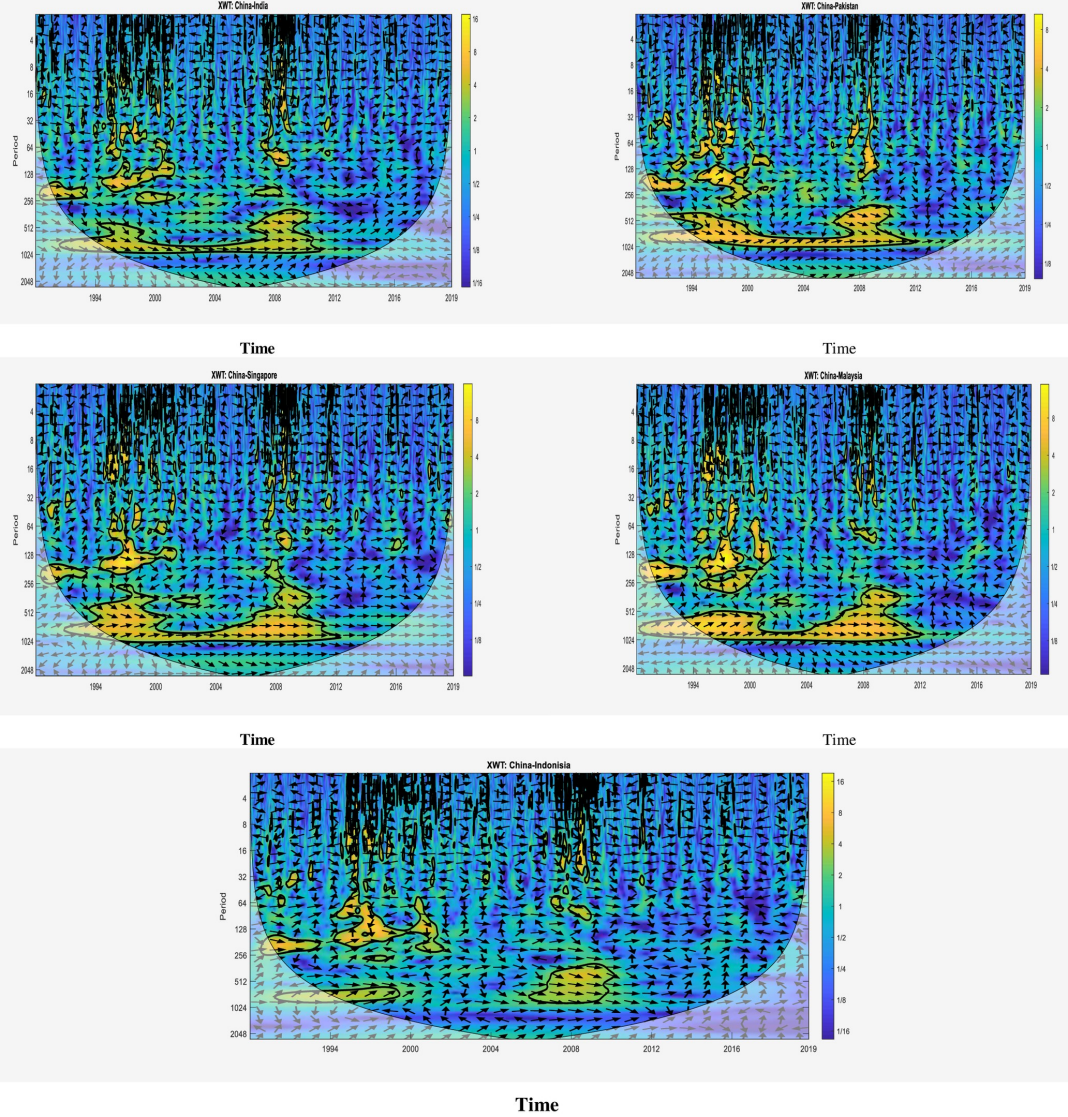
Cross-wavelet power spectra of the emerging stock market. The thick black contour encloses regions where cross-wavelet power spectra is significant at the 5% level against the red noise estimated from Monte Carlo simulations using phase randomized surrogate series. The cone of influence (COI) is indicated by the lighter shade, which delimits the important power regions. The arrows indicate the phase difference between the two time series. The direction of arrows captures the phase difference between two time series. Arrows pointed to the right (left) indicate that variables are in phase (anti-phase), to the right and up (down), the first variable is leading (lagging), and to the left and up (down), the first variable is lagging (leading). Time (year) and frequency (period) are represented on the horizontal and the vertical axis, respectively. The readers can refer to the web version of this article for an accurate interpretation of the graphs.

Cross Wavelet Transform (XWT) technique is used for the characteristic feature extraction where the localized similarities are observed. The advantage of this technique is that it requires fewer number of parameters, compared to the other techniques for time-frame feature classification, to differentiate between normal and abnormal classes. Furthermore, its ability to be compatible with noisy environments allows this technique to obtain more accurate results [83]. It also preserves the information about the phase due to its ability to handle the imaginary part of the input without using the absolute function. Fig 3 represents XWT across the stock market indices of the considered countries. It shall be noted that the arrows indicating phase information help us understand the interrelationships in the variety of different markets. The in-phase relationship in all countries pairs shown in Fig 3, indicated by the right arrow, can be found in numerous significant regions.

Particularly considering, for the China and India couple (Fig 3), the highest level of covariance observed between two-time series and localized at low frequencies, especially during the periods of 1993–2000 and 2007–2008. Generally, in the first (second) periods, the arrows are right up (down). This indicating that two indices are in-phase (in anti-phase) relationship and China stock market indices are leading (lagging). In the case of China and Indonesian’s stock market indices, the two-time series are localized at the highest level of covariance from 1993–2003 and 2007 to 2009. In the first (second) sub-period, the arrows are right and up (down) indicating that the two indices are in-phase and china indices are leading (lagging). Quite similar patterns are found for China–Singapore pair. China-Malaysian covariance pair is high during the period of 1993 to 2000 and 2007 to 2008. In the first (second) period, the arrows are right down (up) which show that the two-series are in-phase and china is lagging (resp. leading). However, China-Pakistan couple is different from other pairs because from 1993 to 2001, over the scale band (128–256) the arrows are right and up showing that china is leading as well as a positive relationship and a high covariance between the two variables, whereas for the frequency band (512–1024), over the most sub-period 1994–2012, the arrows change direction and become right and down. This finding shows that, although the positive relationship between the time series, the Pakistan market is driving the Chine’s one.

When the XWT results of the first period are considered, localized covariance can be observed at low frequencies during the period 1993–2000 with right-up phase directions in case of all pairs. It means that China stock market is in-phase with other markets having by this way a positive relationship with them and leading all these countries in this period. However, this trend changes in the case of the second period 2007–08, where different countries behave in different ways. Stock markets of four out of five countries, i.e., India, Indonesian, Malaysian, and Singapore, are in-phase with China. However, Pakistan’s stock market is an anti-phase relationship with China’s stock market. Also, it can be seen that other than Malaysia and Pakistan, in the remaining markets are led by China’s stock market index. Additionally, high XWT is found for all Asian emerging markets pairs with China in the periods 1993–2000 and 2007–08. Consisting with the findings of [64–66, 68].

The XWT analysis has determined that co-movement of some degree exists between the Chinese stock index and stock indices of Asian emerging economies at short, medium and long horizons in the period 1993 to Aug 2019. In order to validate these findings, further investigations are conducted through Wavelet Coherence (hereafter, WCOH) due to its more authentic results.

### 4.6 Wavelet coherence: China vs. emerging markets

**Fig 4:**
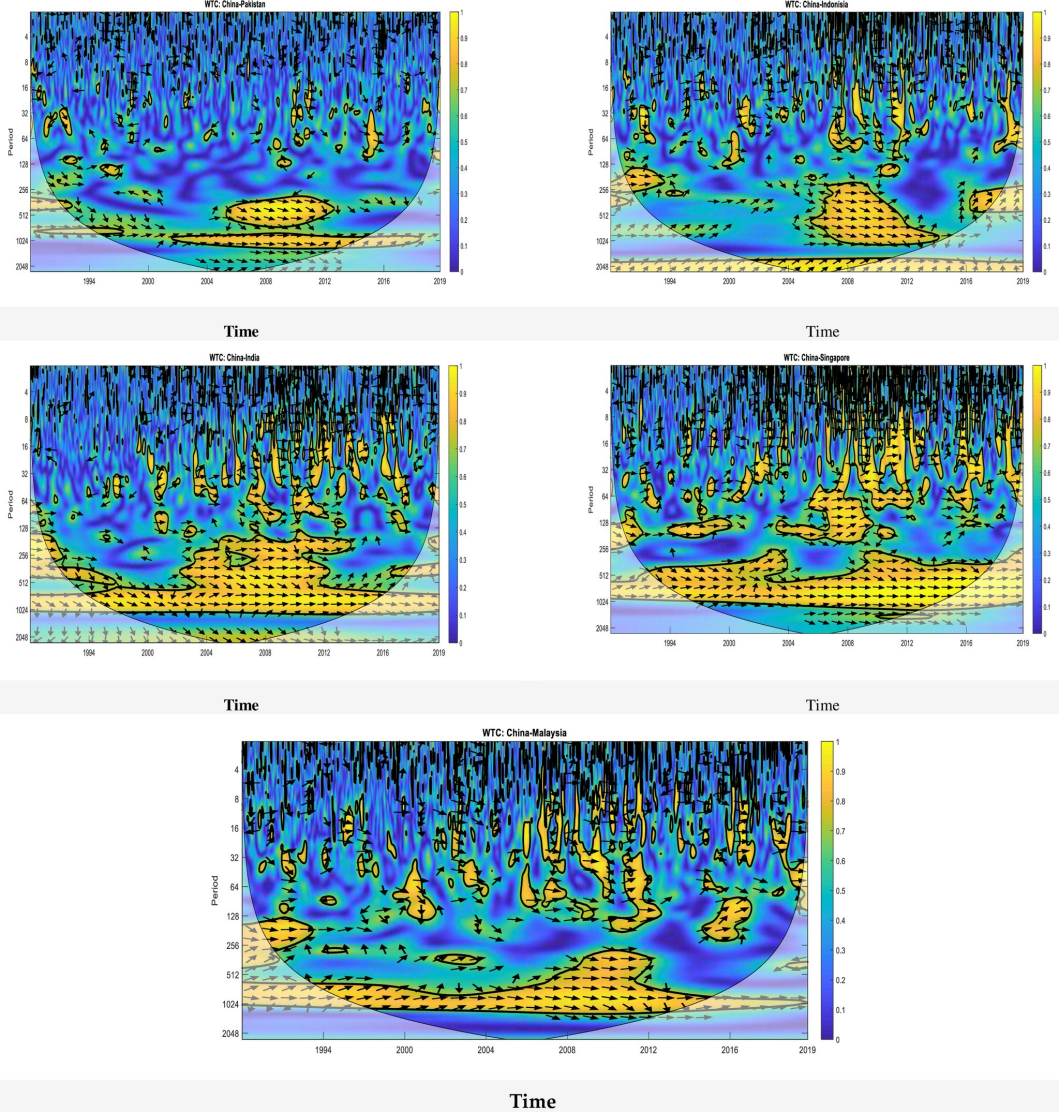
Wavelet coherence of the emerging stock market. The tick black contour encloses regions where the wavelet coherence is significant at the 5% level against the red noise estimated from Monte Carlo simulations using phase randomized surrogate series. The cone of influence (COI) is indicated by the lighter shade which delimits the important power regions. The arrows indicate the phase difference between the two time series. The direction of arrows captures the phase difference between two time series. Arrows pointed to the right (left) indicate that variables are in phase (out of phase), to the right and up (down), the first variable is leading (lagging), and to the left and up (down), the first variable is lagging (leading). Time (year) and frequency (period) are represented on the horizontal and the vertical axis, respectively. The readers can refer to the web version of this article for an accurate interpretation of the graphs.

As defined earlier in section 3.2.4, two-variable WCOH can be stated as a coefficient of localized correlation in time-frequency domain [84]. In order to extract characteristic features in the presence of localized similarities, WCOH techniques are used. Furthermore, these techniques can also be applied to the systems that have a high degree of noise through stock price index baseline correction to achieve higher accuracy. The resulted accuracy from these techniques is relatively good even when it is not possible to fully apply baseline correction. Therefore, WCOH implementation can determine the time-frequency interaction of different counties’ stock markets. The computed cross-wavelet coherence for stock price index pairs in countries of Asia is presented in Fig 4. The theory of convergence of stock price indices across countries in Asia cannot be ignored if large in-phase co-movements are found in the stock price indices of this region. WCOH intensity is represented by color coding (blue to yellow; low coherency to high coherency), where high coherency implies strong correlation. The arrows represent the phase information, where right (left) direction mean in-phase (anti-phase) variables. It can be seen that a huge region of WCOH represents the region of significance. It shall also be noted that most of these phase information (almost majority) represents in-phase stock price indices (right turn arrows) which means that the stock prices of China with other countries are in-phase with a leading effect (Chinese stock prices has a positive causal influence on other countries stocks). Furthermore, high/medium frequency co-movement in most of the countries can be seen. In fact, there are plentiful researches which catch the degree of regional integration among Asian economies increased [85, 86]. Moreover, financial integration and globalization is the retiring trend to stimulate further global connectedness [87].

**Fig 5:**
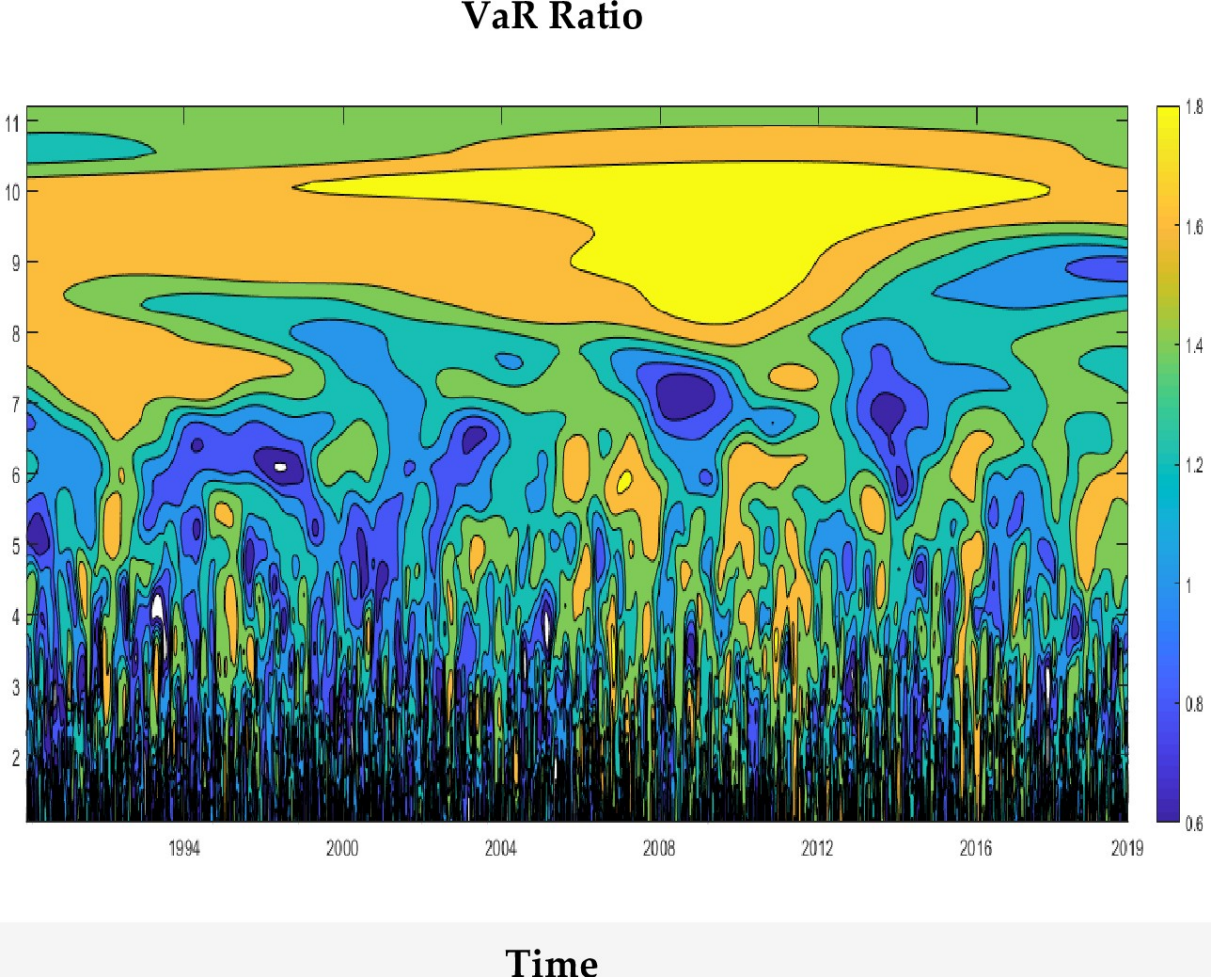
The ratio of the Asian emerging multi-country portfolio variances plot.

The total time period is divided into three parts: 1^st^ (1993–1994); 2^nd^ (2003–2012); and 3^rd^ (2015–2019) in Fig 4. In the first part, five out of five country-pairs, China-India, China-Indonesia, China-Malaysia, China-Pakistan and China-Singapore, show strong low-frequency co-movement in the period 1993–94. In the second part, strong co-movement is observed in the frequency range 256–512 in the period 2003–12 for China-India pair and 2006–12 in China-Indonesia, China-Malaysia, China-Pakistan and China-Singapore pairs, whereas strong co-movement is observed in the frequency range 512–1024 in China-India pair the period 2015–19, China-Indonesia pair in the period 2017–18 and China-Malaysia, China-Pakistan and China-Singapore pairs in the period 2015–16. However, it can be concluded that the stock price indices of China, occurring at a lower frequency, are highly synchronous with the other five counties. A high-level long-run synchronization of the Chinese stock market index with other countries has been detected, which can be seen in Fig 5 where all arrows are right side downwards indicating a cyclic effect where the Chinese stock market is leading (China stock market has a positive influence on other Asian stock markets). In summary, China leads the majority of the Asian countries in the longer run, while slightly leads in shorter run during 2015 and 2016 [88]. Reinhart and Rogoff [89, 90] revealed that all past economic crises share striking similarities in the run-up of debt accumulation, asset prices, current account deficits and growth patterns, although each crisis is characteristically different. Due to the instability created by conflicts such as Kargil, Kashmir, Pakistan-India collisions, Afghan wars and a high degree of terrorism in the region, it is unlikely that this long-term synchronization is enough to drive the conception of an Asian common currency for many years to come.

### 4.7 Value at risk-portfolio diversification: China vs. emerging market

The major goal here is to illustrate the principal findings of our results for portfolio managers of countries in Asia through the VaR. A prominent method to quantify risk at the security level, asset class and portfolio, is named the VaR method. This approach is used to predict the quantity of risk in order to protect the investment funds from exceeding towards the portfolio management caused risk. The present study considers the equally weighted portfolio of multi-country cases from the Asian region. If VaR is at the (1 − *α*)% level of confidence and k denotes the country, and VaR is then defined as:

$$VaR|\alpha |=V_{0}\phi^{-1}(1-\alpha )\phi_{p}$$ (11)

here *V*_0_, *σ*_*p*_, and *Φ* represent initial investment, portfolio returns-variance and the cumulative normal distribution, respectively. It shall be assumed that the portfolio is equally invested in the six considered countries, as practised by [29], the primary reason for a simultaneous investment in six countries would be minimizing the possible risk.

It is important to understand the behavior of other markets if one of them goes down. In other words, it is important to recognize the co-movement among the six considered markets in order to do a safer business. Since the VaR defined in the earlier relationship is the asset mean through portfolio weighted-mean and asset standard derivation through portfolio variance *σ*_*p*_, the k-asset portfolio total risk can be computed as:

$$\phi_{p}^{2}=\sum_{i=1}^{k}\omega_{i}^{2}\sigma_{i}^{2}+\sum_{i}^{k}\sum_{i\neq j}^{k}\omega_{i}\omega_{j}cov(r_{i}r_{j})$$ (12)

here *ω*_*i*_, *r*_*i*_ and *σ*^2^ are stock market weight from the i^*th*^ portfolio, i^*th*^ asset return and estimated variance of the i*th* asset, respectively. The total risk associated with multi-country cases is the sum of the terms: risk at each market; and co-movement degree. The VaR for a portfolio is computed with and without the assumption that no co-movement exists in the markets considered, as practiced in [29, 91, 92]. Eq (12) is used to compute the total risk of the multi-country portfolio for the two cases.

The “portfolio variance ratio” (ratio of co-movement to VaR of the multi-country portfolio) is computed to check whether the co-movement has any effects on the VaR estimations of the multi-country portfolio. When this ratio is equal to 1, none of the quantities is dominant, whereas a ratio higher (lower) than 1 means that VaR (co-movement) is dominant over the other. Eq (9) is used to resort the variances/co-variances counterparts of WCOH, which is then used to compute the “portfolio variance ratio” for the equally-weighted portfolio of the multi-country case. Fig 5 represents the illustrative behavior of this ratio. It is not a surprise that our findings are consistent with the portfolio management theory, where an increase in the risk is expected in case of positively-correlated (over time) portfolio assets. Furthermore, the trend in the effects of co-movements of the Asian stock market on VaR-levels varies throughout the sample period. Nonetheless, the wavelet plot analysis reveals that in the case of lower frequencies, the ratio is higher throughout the sample period, where the VaR is high at almost 75%. Therefore, it can be said that portfolio diversification, in this case, is a good practice. Our findings here are consistent with [29].

Finally, a notable result from this study is that the magnitude of co-movements between the selected markets can influence the rate of VaR of the portfolio. Policymakers and hedge-fund managers, operating in the capital markets of Sothern and South-Eastern Asia, can benefit from these results for more efficient and effective portfolio design.

## 5. Concluding remarks

Due the increasing interest of the investors in emerging economies especially China we analyzed the stock market correlation and volatility of the stock markets of the Asian Emerging economies based on the wavelets methodology. The objective this research is to interrelationship among the selected economies so that the pattern of the relationship between the can be found during the past two decades. we focused specially on China due to its involvement on globl trade. We collected The daily data of Morgan Stanley capital international (MSCI) indices market data over a period of 27 years including the period of global economic crisis period. we selected the stock markets of China, India, Pakistan, Malaysia, Singapore and Indonesia. For each market, the local variance and covariance has been analyzed referring respectively to individual wavelet power spectrum and cross-wavelet transform, and correlation and volatility among the considered markets have been assessed through the wavelet coherence models. China’s stock market has been compared with the other five market indices due to its trade connectedness with the other countries.

As the financial crises strongly affect the co-movement among different countries, which varies both over time and across frequencies. Results show that the co-movement pattern of the considered countries has frequent fluctuations at crisis periods, 1997, 2008 and 2015. This was also observed in the wavelet coherence approach, where change was observed in co-movement at a higher frequency around the periods of the financial crisis. When comparing China stock market with other countries markets at low frequency, a high dynamic correlation (>0.8) is observed in all cases, i.e., India, Indonesia, Malaysia, Pakistan and Singapore. In the case of higher frequencies, high dynamic correlations are observed in different periods for different countries when compared with China. It can be said that the stock market indices of China are synchronous across the considered countries. Moreover, the results of Wavelet Coherence confirm a cyclic effect between Chinese stock market and other five Asian stock markets where China is leading or in another word Chinese stock market has a positive influence on other five stock market prices.

The results of the preliminary analysis also showed that there are co-movement patterns of higher frequencies during the crisis’s periods of 1997, 2008, and 2015. The closeness among the stock markets considered economies show that the crisis period converged the economies reducing the differentials of the return on the stock markets of the Asian emerging economies as found by [4]. The closeness of the stock market movements show the integration of the stock markets thus bringing these economies closer. Similarly, the sporadic movements during the normal periods show that the stock markets have dissimilar returns which contains opportunity for the international stock markets investors willing to spread their investment in dissimilar ass et so that the risk can be reduced [93].

In general, the results show that same risk pattern during the selected sampling period with the exception of Malaysia and India. The riskiest market of these domains corresponds to the Singapore market, where a large yellow island was distributed in the 1994–2012. This indicates that recent global financial crises have greatly affected on Asia's emerging markets. However, a high variation, in the low frequency for Malaysian stock indices during 2007–08 can be seen in longer horizons, which is not the same for other countries considered. In past two decades the economic development and increasing trade of China around the world has started affecting the its trade partners. The synchronization of Chinese economy with the other countries is due to it trade association with the Asian emerging economies. However, due to the recent conflicts of China with USA, and India can pose a risk to the closeness of China with other countries specially its neighboring East Asian countries. Similarly, the other counties can also play role in the economic integration with better performing economies like China which will have positive impact on their economies.

In a financial point of view, the increase in coherency among the Asian emerging economies during financial crisis periods suggest that verifies the contagion hypothesis. From an empirical point of view, the variation of the correlation coefficient with respect to time suggests structural breaks in the asset price at the time of the crises. This research can help portfolio managers operating in this region to gather possible consequences, and they are encouraged to analyze co-movements with respect to both frequency and time, modern portfolio theory offers a formal framework to consider portfolio in the time domain, it can be an insight for further research in this area. Additionally, the outcomes also have valuable implications for policymakers in the context of China stock markets with the neighboring countries. This research can help portfolio managers for mitigating global risk and transaction risk, governments for refining macroeconomic policies and for avoiding financial distress through intervention, and individual financier for creating diversified portfolios and enhancing profits.

## Supporting information

S1 Appendix Selected countries and their indices. (DOCX)
