# Supplementary material for: Stock market comovements among Asian emerging economies: A wavelet-based approach
Source: PLoS One. 2020 Oct 12;15(10):e0240472. doi: 10.1371/journal.pone.0240472 (PMC7549817; doi:10.1371/journal.pone.0240472)
Supplement: S1 Appendix — (DOCX) [file pone.0240472.s001.docx]

**S1 Appendix.** Selected countries and their indices.

| Stock Market | Symbols | Benchmark Indices |
| --- | --- | --- |
| China | CHIN | MSCI China index |
| India | IND | MSCI India index |
| Indonesia | INDO | MSCI Indonesia index |
| Malaysia | MAL | MSCI Malaysia index |
| Pakistan | PAK | MSCI Pakistan index |
| Singapore | SING | MSCI Singapore index |
